# Amputations secondaires du membre après une chirurgie primaire des fractures ouvertes du membre inferieur

**DOI:** 10.11604/pamj.2018.29.172.13177

**Published:** 2018-03-26

**Authors:** Kouamé Innocent M’Bra, Aya Adélaïde Natacha Kouassi, Bada Justin Leopold Niaore Sery, Loukou Blaise Yao, Kouamé Jean Eric Kouassi, Pierre Germain Ochou, Yao Aboh Ganyn Robert Arnaud Asséré, Grah Franck Lohourou, Koffi Léopold Krah, Michel Kodo

**Affiliations:** 1 Service d’Orthopédie et de Traumatologie du CHU de Bouaké, Côte d’Ivoire

**Keywords:** Amputation secondaire, fracture ouverte, gangrène infectieuse, gangrène ischémique, ostéite, Secondary amputation, open fracture, infectious gangrene, ischemia gangrene, osteitis

## Abstract

Les auteurs rapportent les complications survenues au cours de la prise en charge des fractures ouvertes de membres inférieurs, observées ces dernières années dans leur pratique et qui ont abouti à des amputations secondaires, tout en analysant les différents facteurs de risque et les déficits éventuels dans la prise en charge qui ont pu être à l'origine de cette évolution. Il s'agissait d'une étude rétrospective s'étendant sur une période de 06 ans ( Janvier 2006 - Janvier 2012). Elle concernait des patients ayant présenté une fracture ouverte de membre inférieur traité initialement dans notre institution et dont les suites se sont compliquées et ont nécessité une amputation. Tout patient dont l'amputation avait été réalisée d'emblée après l'examen à l'entrée aux urgences était exclu. Egalement tout patient traité dans un autre hôpital avant d'être admis chez nous était exclu de l'étude, même si une amputation secondaire avait été réalisée dans notre institution. Nous avons récolté les différentes données en analysant les dossiers de patients (examens cliniques et complémentaires, comptes rendus opératoires). Nous avons évalué notre prise en charge en fonction des différents guidelines et recommandations retrouvés dans la littérature. Ces complications ont été observées chez neuf patients sur les 306 fractures ouvertes de membre inférieur prises en charge sur la même période (Janvier 2006 - Janvier 2012), soit 2,9 . L'âge moyen était de 42,6(26-57) ans, la totalité des patients était de sexe masculin. Nous avons enregistré 1 cas de fracture du fémur, 7 cas de fracture des 2 os de la jambe et 1 cas d'écrasement du pied. Il s'agissait de fracture ouverte dont 1 cas de type 1; 3 cas de type II et 5 cas de type III selon la classification de Gustilo et Anderson. 5 cas d'amputations à la cuisse ont été réalisées et 4 cas d'amputation au tiers supérieur de la jambe. Les différentes complications ayant motivées ces amputations étaient des infections osseuses ou des gangrènes de parties molles d'origine ischémique ou infectieuse. Les patients n'avaient pas de tares favorisantes telle que le diabète et n'étaient pas tabagiques chroniques. Nous n'avons pas constaté de décès. Les fractures ouvertes méritent une attention particulière du chirurgien tant au point de vu diagnostic des complications immédiates que thérapeutique afin de réduire l'incidence des amputations post chirurgie primaire donnant l'impression au patient d'avoir été initialement mal opéré. Une attention particulière doit être portée au degré de contamination initiale et à la présence d'un germe virulent sur le lieu du traumatisme, pouvant motiver des attitudes particulières lors de la prise en charge primaire.

## Abstract (en)

This study reports the complications occurred during the management of open fractures of the lower limbs, resulting in secondary amputations, observed in clinicians' practice in recent years as well as different risk factors and possible deficiencies in management at the origin of these complications. We conducted a retrospective study over a period of 06 years (January 2006 - January 2012). It included patients with open fracture of the lower limb initially treated in our institution and whose complications resulted in amputation. All patient undergoing emergency amputation after examination at the Emergency Department were excluded. All patient treated in another hospital before being referred to us were excluded by the study, even if secondary amputation had been performed in our institution. We collected data by analyzing the records of patients (clinical and complementary examinations, surgical reports). We evaluated our management of open fractures of the lower limb according to the guidelines and recommendations found in the literature. These complications were observed in 9 out of 306 open fractures of the lower limb treated in the same period (January 2006 - January 2012), reflecting a rate of 2.9%. The average age was 42.6(26-57) years, all patients were male. We recorded 1 case of fracture of the femur, 7 cases of fracture of 2 leg bones and 1 case of foot crushing. These were open fractures including 1 case of type 1 fracture, 3 cases of type II fracture and 5 cases of type III fracture, according to Gustilo and Anderson classification. We performed 5 amputations of the thigh and 4 amputation of the upper third of the leg. Various complications motivated these amputations, including bone infections or soft-tissue gangrenes of ischemic or infectious origin. The patients had no morbidities such as diabete and were not chronic smokers. No patient died. Open fractures deserve special attention of the surgeon on immediade complications both from a diagnostic and therapeutic point of view in order to reduce the amputation rates after primary surgery giving patients the impression that they had an incorrect procedure performed on them. Particular attention should be paid to the degree of initial contamination and to the presence of a virulent germ at the site of trauma which may motivate particular attitudes during primary management.

## Introduction

Les fractures ouvertes de membre présentent une fréquence relativement élevée et leur survenue est majoritairement liée aux accidents de la voie publique [1]. Elles touchent différentes composantes osseuses de l'organisme et exposent à de nombreuses conséquences entre autres les amputations de membre. Quand on sait que les amputations d'origine traumatiques représentent environ 7 des étiologies des amputations [2]. On note une tendance croissante des tentatives de sauvetage initial du membre traumatisé à tout prix, face à des lésions cliniques et radiologiques importantes, devant le refus tout à fait compréhensif et justifié du patient de se faire amputer . Le terme d'amputation secondaire prend évidemment donc de l'ampleur .Il serait donc intéressant identifier les facteurs qui pourrait être amélioré et aussi évaluer la qualité de la prise en charge qui reste déficiente selon certains auteurs [3] au vu des différents guidelines présents dans la littérature et qui ne sont pas toujours entièrement appliqués. Ici nous rapportons des cas d'amputations secondaires de membre que nous avons rencontré dans notre pratique et analysons les différents facteurs de risque et les déficits éventuels dans la prise en charge qui auraient pu être à l'origine de cette évolution.

## Méthodes

Il s'agissait d'une étude rétrospective s'étendant sur une période de 06 ans ( Janvier 2006 - Janvier 2012) et qui s'est intéressée à neufs patients représentant 2,9% des 306 patients reçus pour fracture ouverte du membre inférieur au cours de la même période. Il s'agissait de patients ayant présenté une fracture ouverte de membre inférieur traité initialement dans notre institution et dont les suites se sont compliquées et ont nécessité une amputation. Tout patient dont l'amputation avait été réalisée d'emblée après l'examen à l'entrée aux urgences était exclu. Egalement tout patient traité dans un autre hôpital avant d'être admis chez nous était exclu de l'étude, même si une amputation secondaire avait été réalisée dans notre institution. Nous avons récolté les différentes données par analyse des dossiers de patients (examens cliniques et complémentaires, comptes rendus opératoires). L'âge des patients, le mécanisme de survenue, la gravité de la lésion selon la classification de Gustilo et Anderson [**4**], le traitement médical administré, le délai de la chirurgie; calculé entre l'admission du patient à l'hôpital et le début de la chirurgie comme marqué sur la fiche anesthésique, les gestes chirurgicaux, les complications, le nombre de réintervention, le délai pour l'amputation et le niveau d'amputation étaient les différents paramètres analysés. Nous avons évalué notre prise en charge en fonction des différents guidelines et recommandations retrouvés dans la littérature.

## Résultats

Tous nos patients étaient de sexe masculin d'âge moyen de 42,6(26-57)ans, exerçant tous dans le secteur tertiaire. Le délai moyen de la prise en charge était de 1,1 jour (8h-3jours). Six cas de fracture de jambe sont survenus au cours d'un accident de la voie publique, 5 parmi ces patients étaient des piétons et 1 était passager. 1 cas de fracture du fémur est survenu au cours d'une chute à moto. Un cas de fracture de jambe au cours d'un accident champêtre, le patient aurait reçu une branche d'arbre sur la jambe. Un écrasement du pied était retrouvé chez un patient qui aurait eu le pied encastré dans un engrenage sur une plateforme industrielle. Le patient ayant présenté la fracture du fémur a été admis environs 8 heures après le traumatisme. Il présentait une fracture supracondylienne ouverte assimilée au type II selon la classification de Gustilo et Anderson, sans complications vasculaire ni nerveuse observées à l'admission, la prise en charge chirurgicale a été faite à plus de 24 heures après son admission et a consisté à un parage et une contention par fixateur externe, sous anesthésie générale. Le traitement médical a été réalisé selon notre protocole habituel et consistait en une administration d'antibiotique pré et post opératoire: 2 g par jour de ceftriaxone, 500 mg trois fois par jour de métronidazole par voie intraveineuse, une prophylaxie antitétanique à la dose de 1500 UI de sérum antitétanique, de l'héparine de bas poids moléculaire 40 mg par jour en sous-cutané en traitement préventif des complications thromboemboliques, des antalgiques à la demande. A noté après cinq jours d'hospitalisation une gangrène sèche du pied a été constaté. A l'origine une embolie vasculaire. Une amputation au tiers supérieur de la jambe a été réalisée. Deux cas de fractures de jambe grade 1 et 3a de Gustilo survenues au cours d'un accident de la voie publique ont bénéficié du protocole médical, d'un parage et d'une ostéosynthèse par fixateur externe. Nous avons constaté deux jours plus tard la survenue d'une gangrène humide remontant au tiers supérieur de la jambe, ce qui a nécessité une amputation à la cuisse. Un cas de fracture ouverte de jambe type 3b a été médicalement traité selon notre procédure médicale, puis parage et stabilisation par fixateur externe. La complication s'est faite sous forme d'une ostéite chronique massive du tibia qui a abouti après plusieurs chirurgies de sauvetage à une amputation à la jambe au tiers supérieur un (1) an plutard. Les autres cas de fracture de jambe type 2 et 3b médicalement traité selon le protocole habituel se sont compliquées après un parage et une contention par fixateur externe ou un enclouage centromédullaire pour d'autres, d'une gangrène gazeuse ce qui a nécessité une amputation dans trois cas à la cuisse au tiers inférieur et un cas à la jambe au tiers supérieur. Quant à l'écrasement du pied un parage et une contention dans une attèle postérieure ont été réalisé. En peropératoire on a mis en évidence un important délabrement de l'artère pédieuse et l'artère tibiale postérieure n'a pas été explorée. Les suites se sont compliquées d'une gangrène humide du pied et une amputation à la jambe a été réalisée au tiers supérieur en vue d'un appareillage adéquat (Tableau 1).

**Tableau 1:** Récapitulatif des caractéristiques étiologiques, lésionnelles et évolutives

Patients	Etiologie	Segment traumatisé	Grade Gustilo et Anderson	Délai chirurgie (heure)	Procédure chirurgie primaire	Motif d’amputation	Délai d’amputation	Niveau d’amputation
**Patient 1**	AVP	Jambe	1	˃ 6	Parage + clou	Gangrène infectieuse	6 jours	cuisse
**Patient 2**	AVP	Jambe	2	˃ 6	Parage + clou	Gangrène infectieuse (gazeuse)	2 jours	jambe
**Patient 3**	AVP	Jambe	2	˃ 6	Parage + clou	Gangrène infectieuse (gazeuse)	2 jours	cuisse
**Patient 4**	AVP	Jambe	3a	˃ 6	Parage + fixateur externe	Gangrène infectieuse	3 jours	cuisse
**Patient 5**	AVP	Jambe	3b	˃ 6	Parage + fixateur externe	Ostéïte chronique	1 an	jambe
**Patient 6**	AVP	Jambe	3b	˃ 6	Parage + fixateur externe	Gangrène infectieuse (gazeuse)	3 jours	cuisse
**Patient 7**	AVP	Cuisse	2	˃ 6	Parage + fixateur externe	Gangrène vasculaire	5 jours	jambe
**Patient 8**	accident de travail	Jambe	3b	˃ 6	Parage + fixateur externe	Gangrène infectieuse (gazeuse)	3 jours	cuisse
**Patient 9**	accident de travail	Pied	3C	˃ 6	Parage + attèle	Gangrène infectieuse	5jours	jambe

**AVP :** accident de la voie publique

## Discussion

La prise en charge des fractures ouvertes constitue un challenge pour tout chirurgien traumatologue face aux multiples complications que cela pourrait engendrer tant sur le plan infectieux, vasculaire ou cutané avant même les aléas de la consolidation. Cela demeure toujours un sérieux problème de santé publique qu'il ne faudrait pas occulter bien qu'on ait enregistré d'important progrès au plan décisionnel et thérapeutique au vu des différents scores et guidelines décrits dans la littérature [5, 6]. La proportion de patients secondairement amputés dans notre contexte (1,5 cas / an) est fort élevée pour nous car cet évènement reste tragique et difficile à supporter pour l'individu concerné et même pour son entourage. Un audit donc de notre pratique et de nos protocoles s'avère nécessaire afin de contribuer à la régression voir à annihiler cette évolution qui apparait souvent comme un échec de la prise en charge. Au plan épidémiologique l'âge moyen de 42,6 ans représente une population jeune active et ne présentant pas de tares majeures donc pourvue d'une évolution favorable. Toutes ces fractures sont survenues dans un cadre de haute vélocité comme cela est décrit dans la littérature [1] exposant à des lésions importantes des parties molles et osseuses retenues comme un critère péjoratif dans le MESS (mangled extremity severity score) [5]. Helfet [5] auteurs du score prédictif MESS, soutenus par d'autres auteurs [7, 8] suggèrent que toute valeur inférieure à 7 doit faire indiquer une conservation du membre et toute autre valeur supérieure ou égale à 7, faire poser l'indication d'une amputation. Plusieurs auteurs considèrent que des valeurs élevées de ce score (MESS), supérieures ou égales à 7 ne doivent pas être considérée comme indicatrices d'une sanction radicale [9]. Tandis même que certains auteurs s'opposent à l'utilité de tel score pronostic [10]. Bosse [11] démontre qu'un score bas peut suffisamment prédire une conservation du membre par contre un score élevé supérieur ou égale à 7 ne prédit en rien le devenir du membre. L'écrasement du pied et celui de la jambe au cours d'un accident champêtre observés dans notre contexte présentaient un MESS élevé (>7) et présageait d'une évolution pas tout à fait rassurante devant surtout la lésion de l'artère pédieuse dans le premier cas. Mais nous avons été motivés pour la tentative de sauvetage en espérant une suppléance de l'artère tibiale postérieure.

Les amputations pour la majorité des cas de notre série étaient dues à des infections sur des fractures qui présentaient des scores (MESS) inférieurs à 7. Bien que le protocole médical habituel pratiqué dans nos habitudes soit respecté chez ces 9 patients. Ce protocole est sensiblement superposable au guideline proposé par le BOA / BAPS (British Orthopaedic Association/British Association of Plastic Surgeons) [6]. A noter que ces infections retrouvées étaient dues à des germes anaérobiques (4 cas de gangrènes gazeuses). Les fractures seraient survenues sur des terrains non recouverts qui sont de véritables réservoirs de spores de clostridiums perfringens retrouvés habituellement dans la gangrène gazeuse. Dans tous les cas, même si une éventuelle contamination nosocomiale était exclue, on n'occulterait pas d'incriminer un parage insuffisant. Vu que la majorité des patients ont été pris en charge en dehors des heures normales de travail précisément les nuits ce qui pourrait contribuer à la baisse de la performance dans la prise en charge. Nos délais pour la chirurgie primaire doivent être considérablement revu à la baisse. Aucun des patients n'a été opéré avant le délai classique de 6 heures recommandé. Quand à ce patient présentant une ostéite massive, il est sans nul doute qu'elle provienne d'une infection nosocomiale vu le caractère multi résistent du germe rencontré. L'isolement, en salles régulièrement stérilisé, des patients présentant une fracture ouverte s'avère être une solution pour palier à ce type de complication. Le choix du niveau d'amputation a été influencé par le niveau supérieur de la lésion dans l'optique d'optimiser les chances de guérison bien que nous ayons à l'esprit de maintenir la fonctionnalité optimale du membre concerné Au cours des tentatives de sauvetage du membre, la sélection des patients est d'une importance majeure. Au tout début le patient doit réaliser que la fonction et l'apparence du membre ne seront pas complètement restaurées comme avant l'accident. La tentative de sauvetage peut conduire à un sepsis, une ostéite, une amputation secondaire et dans des cas plus dramatiques à la mort du patient. Il existe néanmoins des cas ou le membre a pu être sauvé bien que le contexte prédisait une évolution défavorable, ce qui a également été observé dans la littérature [9].

## Conclusion

Les fractures ouvertes méritent une attention particulière du chirurgien tant au point de vu diagnostic des complications immédiates que thérapeutique afin de réduire l'incidence des amputations post chirurgie primaire donnant l'impression au patient d'avoir été initialement mal opéré. Le tout réside dans un examen soigneux du malade même si les conditions sont rendues difficiles par le contexte de traumatisme multiple du patient difficile à mobiliser et souvent dans un cadre de patients multiples à examiner aux urgences, le médecin se retrouve donc débordé. Dans tous les cas, l'examen doit être complété sur la table opératoire chez un patient sous anesthésie locorégionale ou endormi garant donc d'un choix décisionnel thérapeutique optimal. Toute tentative acharnée, raisonnable de conservation du membre n'est pas pour nous à décrier mais il faudra expliquer clairement au patient conscient, l'objectif qu'on chercherait à atteindre et les différents résultats qu'on pourrait obtenir.

### Etat des connaissances actuelle sur le sujet

- Selon la littérature les importants defects de tissus mous et osseux, les lésions vasculaires et les lésions ipsilatérales sont considérés comme des facteurs prédictifs d'une amputation secondaire du membre;
- Existence de guidelines contribuant à réduire efficacement les complications dans la prise en charge de fracture ouvertes.

### Contribution de notre étude à la connaissance

- On observe des possibilités de survenue d'infection grave malgré le bas grade de l'ouverture cutanée. Tenir donc compte du degré de contamination initiale qui pourrait influencer sensiblement l'évolution de la lésion;
- Le parage des fractures ouvertes doit être réalisé avec autant de précaution pour les ouvertures de bas grade comme celui des hauts grades.

## Conflits d’intérêts

Les auteurs ne déclarent aucun conflit d'intérêts.
